# Morpho-Anatomical and Physiological Responses Can Predict the Ideal Period for the Transplantation of Hydroponic Seedlings of *Hymenaea courbaril*, a Neotropical Fruit Tree

**DOI:** 10.3390/plants9060721

**Published:** 2020-06-06

**Authors:** Daniele de Cássia Vieira de Sousa, Layara Alexandre Bessa, Fabiano Guimarães Silva, Márcio Rosa, Sebastião de Carvalho Vasconcelos Filho, Luciana Cristina Vitorino

**Affiliations:** 1 Laboratory of Plant Mineral Nutrition, Instituto Federal Goiano, Rio Verde Campus, Rodovia Sul Goiana, Km 01, Rio Verde 75901-970, GO, Brazil; daniele.de@hotmail.com (D.d.C.V.d.S.); layara.bessa@ifgoiano.edu.br (L.A.B.); fabiano.silva@ifgoiano.edu.br (F.G.S.); 2 Fazenda Fontes do Saber, Rio Verde Campus, Universidade de Rio Verde (UniRV), Caixa Postal 104, Rio Verde 75901-970, GO, Brazil; marcio.rosa@ifgoiano.edu.br; 3 Laboratory of Plant Anatomy, Instituto Federal Goiano, Rio Verde Campus, Rodovia Sul Goiana, Km 01, Rio Verde 75901-970, GO, Brazil; sebastiao.carvalho@ifgoiano.edu.br; 4 Laboratory of Agricultural Microbiology, Instituto Federal Goiano, Rio Verde Campus, Rodovia Sul Goiana, Km 01, Rio Verde 75901-970, GO, Brazil

**Keywords:** hydroponics, chlorophyll *a* fluorescence, gas exchange, stomatal density

## Abstract

Hydroponics is an excellent alternative approach for the production of seedlings, given the growing demand for fruiting trees for the reforestation or recuperation of degraded natural landscapes. In most cases, however, little is known about the optimal period for the maintenance of the seedling in the hydroponic system. Given this, we decided to investigate the hypothesis that morpho-anatomical and physiological alterations can be used to predict the optimal timing for the transplantation of the seedlings to the soil substrate, thereby guaranteeing the most cost-effective application of the hydroponic system. We selected *Hymenaea courbaril* L., an important Neotropical fruit tree, as the model for this study. We cultivated *H. courbaril* seedlings in a static hydroponic system and evaluated morpho-anatomical, physiological, and growth parameters over the course of seedling development (30, 60, 90, 120, 150, and 180 days after transplantation; DAT). We observed an interesting relationship between the increase in the density (SD) and conductance (*gsw*) of the stomata up to 120 DAT, which reflected higher rates of photosynthesis (*A*), but also a reduced efficiency in the use of water. In the subsequent intervals, the SD of the plants and the diameter of the radicular xylemic vessels elements (RVE) decreased, in an attempt to increase the efficiency of the use of this resource. We also observed an increase in the thickness of the palisade parenchyma (PP) prior to 120 DAT, which did not reflect a general increase in the thickness of the mesophyll, indicating an adjustment in the thickness of the spongiform parenchyma (SP). We also observed a progressive increase in photosynthetic efficiency up to 120 DAT, based on parameters such as the absorption flux energy per active reaction center (ABS/RC) and the photosynthetic performance index (PI_ABS_), but after this period these indices decreased progressively. However, as the PI_ABS_ is an indicator of the plant’s tolerance, its decline was associated with an increase in the dissipation of energy (DI_0_/RC), which indicates that, after 120 DAT, the plant pots may become a stress factor that limit the growth of *H. courbaril* seedlings. The results of the present study indicate conclusively that a 120-day period is the optimum for the maintenance of the *H. courbaril* seedlings in the hydroponic system, and also confirm the hypothesis that the morpho-anatomical and physiological responses observed in the plants can be used to predict the ideal period for the transplantation of the seedlings, contributing to a reduction in production time of the hydroponic system.

## 1. Introduction

Worldwide, there is a growing need to increase and diversify the production of seedlings for both conventional agriculture and the recuperation of areas of natural vegetation that have been degraded by human activities. One potential solution for this problem has been the adoption of hydroponics, which has been used increasingly for the large-scale production of seedlings, which can be obtained in less time than traditional techniques, as well as providing more effective control of pests and disease that may damage the plant in the initial stages of development and have negative repercussions for its later growth [1]. Hydroponic systems also provide greater control and precision in the supply of water and nutrients, and the management of the culture, including the potential for the reuse of the nutrient solution, which not only permits the maximization of resources, but also the reduction of the lixiviation of fertilizers to aquatic ecosystems, in comparison with the direct fertilization of the soil [2].

Even so, hydroponics has a number of potential limitations, including the need for specialized technicians, in particular, for the maintenance and running of dynamic and complex systems, such as aeroponics [2,3], and requires substantial knowledge and experience in the development of seedlings in this type of system. The definition of the maximum amount of time that a seedling should remain in the system, for example, is fundamentally important to avoid excessive plant maintenance costs. On the other hand, removing the seedling from the system prematurely may risk interrupting its development and threatening the subsequent survival of the plant. However, we do know that plants under water, saline, or alkaline stress, and even the effects of metals, may present specific morpho-anatomical and physiological alterations [4,5,6], which may either manifest this stress or represent adaptive adjustments [7]. These alterations range from shifts in the density of stomata and their conductance to the accumulation of osmoprotectants or phytohormones [8,9]. But, is it possible that seedlings that are sufficiently developed will begin to treat the hydroponic system as a limiting factor for their development? In response to this question, we decided to test the hypothesis that specific morpho-anatomical and physiological responses from the plant may signal the passing of the ideal period for the transplantation of seedlings from the hydroponic system to the soil. To test this hypothesis, we chose *Hymenaea courbaril* L., a fruit-producing tree species that is amply distributed in the Neotropical region [10], and may be tolerant of a wide range of environmental conditions [11]. Given these characteristics, this species is a promising candidate for reforestation programs or the regeneration of natural habitats [12], and it has been included in the 25 priority species for recuperation programs of areas of degraded forest [13].

*Hymenaea courbaril* is a climax species, whose growth is limited by low light levels [14]. Given this, the seedlings of this species tend to grow slowly during their initial development under natural conditions, which exposes the plant to abiotic stressors and predation, and tends to reduce seedling recruitment during recolonization. Many studies have provided evidence in favor of the use of hydroponic systems as a viable alternative for the mass production of the seedlings of native fruit trees such as *H. courbaril* (e.g., [15,16,17,18,19]), in particular, by optimizing their initial growth. But as this system requires a considerable financial investment and has high running costs, minimizing the period of hydroponic cultivation is important to control costs for the producer.

Techniques such as the measurement of concentrations of photosynthetic pigments and the nutrient content of the soils and culture media are now common in the field, given that many laboratories now provide easy access to the classical methods for the determination of nutrient concentrations and versatile portable equipment for the rapid quantification of photosynthetic pigments and even the levels of nitrogen and flavonoids found in the leaves (see [20,21]). It is thus possible that, in the future, the understanding of specific anatomical modifications and physiological parameters related to chlorophyll *a* fluorescence and gas exchange can be used as cues for the recognition and assessment of the occurrence of stressful conditions that can affect productivity [22,23], contributing to the development of optimal systems for the growth and maintenance of seedlings. Carstensen et al. [24] demonstrated that the analysis of chlorophyll *a* fluorescence can detect phosphorus deficiencies under field conditions, providing an effective tool to ensure the productivity of barley, *Hordeum vulgare* L.

Chlorophyll *a* fluorescence provides detailed evidence on the absorption status and use of light energy by the reaction centres of photosystem II (PSII), and can also detect possible damage to the photosynthetic apparatus, providing parameters for the monitoring of the physiological conditions of plants under abiotic stress [25]. Gas exchange parameters can also provide insights into situations of stress, which may result in a reduction in productivity (e.g., [26,27,28]). As the methods used to measure fluorescence and gas exchange are non-invasive and non-destructive, they can be used to monitor the plant over long periods, providing more reliable inferences for the testing of hypotheses [29]. The current availability of portable devices that measure parameters of fluorescence and photosynthesis has expanded significantly the potential for the application of this approach for the monitoring of plantations (see [30,31]). As the anatomical techniques require only small samples of tissue and are well established (see [32]), the present study evaluated whether these techniques, combined with the parameters of fluorescence and gas exchange can be used to identify the ideal period for the transplantation to the soil of the seedlings of fruit trees, such as *H. courbaril*, raised in hydroponic systems.

## 2. Material and Methods

### 2.1. Acquisition of the Seedlings and Cultivation Conditions

The experiment was conducted in a greenhouse of the Plant Mineral Nutrition Laboratory of the Rio Verde Campus of the Federal Institute of Goiás (17°48′15.9” S, 50°54′19.5” W) between January and December 2019. The *H. courbaril* fruits were collected at Varginha Farm (17°32′48.66” S, 50°50′11.71” W), in the municipality of Rio Verde, Goiás, Brazil. The fruit were washed and the pulp was removed manually under running water. The seeds were scarified mechanically to break their dormancy, with the side opposite the hilum being rubbed with sandpaper until the tegument was abraded visibly. The seeds were then sown in plastic trays containing a substrate of autoclaved sand.

Sixty days after sowing, when they presented 3 or 4 well-defined leaves, the seedlings were transplanted to 8-L hydroponic pots. The seedlings were then maintained for 15 days in half ionic strength Hoagland & Arnon [33] nutrient solution. Following this adaptation, the plants were exposed to the full-strength solution. The plants were cultivated in a nutrient solution under a mean irradiance of 400 μmol m^−2^ s^−1^, under constant aeration. The pH was adjusted to 5.5 ± 0.5 through the addition of HCl or NaOH, as necessary, and the nutrient solution was changed whenever there was a 30% depletion of the initial electrical conductivity.

The plants were evaluated at 30-day intervals starting on the Date of Transplantation (DAT), that is, at 30, 60, 90, 120, 150, and 180 DAT, when morpho-anatomical, physiological, and growth data were collected. The experiment was conducted in random blocks, with the data being collected in eight repetitions (one repetition = a pot containing two plants) per evaluation period (treatment). Thus, 16 experimental units were evaluated in each sampling period.

### 2.2. Morpho-Anatomical Evaluations

At 30, 60, 90, 120, 150, and 180 DAT, 0.5 cm^2^ samples were collected from the middle third of the leaves using disposable razor blades. These samples were fixed in Karnovsky [34] solution for 24 h, dehydrated in an increased series of ethanol, and pre-infiltrated and infiltrated in historesin (Historesin-Leica), following the manufacturer’s protocol. Traverse sections, 5 µm thick, were then obtained in a rotary microtome (Model 1508R). The sections were stained with 0.05% toluene blue (polyacromatic toluidinacoloration) in 0.1 M phosphate buffer, at pH 6.8 for structural and micromorphometric analyses [35]. The sections were obtained from one plant per repetition.

The thickness of the palisade parenchyma (PP) and the spongiform parenchyma (SP) were determined using the ImageJ (Open source code—http://rsbweb.nih.gov/ij/download.html) image processing software [36]. As *H. courbaril* is considered to be hypostomatal [37]; the stomatal density (SD, stomata mm^−2^) was determined from 30 observations per plant of the abaxial surface of the epidermis, covering a total field of 0.067 mm^2^. The diameter of the xylemic vessels elements of the stem (SVE) and root (RVE) were measured from transverse sections obtained manually using a razor blade. These sections were stained with lignin with phloroglucinol acid and the measurements were taken within an area corresponding to 25% of each transverse section. The SD and vessel diameters were measured using the Image-Pro Plus 4.5 software (Media Cybernetics, Silver Spring, MD, USA).

### 2.3. Physiological Evaluation

The photosynthetic pigments were evaluated through the examination of three leaf discs, 5 mm in diameter, obtained from all the sample units. These discs were incubated in a solution of dimethylsulfoxide (DMSO) saturated with CaCO_3_ [38], stored in sealed tubes wrapped in aluminum foil for 24 h at 65 °C. The absorbency of the extract was determined by spectrophotometry, using wavelengths of 665, 649, and 480 nm. The concentrations of chlorophyll *a* (Chla), chlorophyll *b* (Chlb), total chlorophyll (TChl), and carotenoids were estimated based on [39].

The chlorophyll *a* fluorescence OJIP transient was determined using a portable FluorPen FP 100 fluorometer (Photon Systems Instruments; Drasov, Czech Republic). The fourth leaf of all the sample units was first adapted to the dark for 30 min for the complete oxidation of the photosynthetic system of electron transportation. The leaves were then exposed to a 3000 µmol m^−2^ s^−1^ pulse of blue light, with the minimum fluorescence (F_0_) being measured at 50 μs when all the PSII reaction centers are open, and defined as step O, followed by step J (at 2 ms), step I (at 30 ms), and the maximum fluorescence (F_M_) when all the PSII reaction centers are closed, which is known as step P. These values were used to estimate the different bioenergetic indices of the PSII, following [40], that is, the absorption flux energy per active reaction center (ABS/RC), the energy flux captured by each reaction center (TR_0_/RC), the electron transport flux per reaction center (ET_0_/RC), the specific flux of the dissipation of energy at the level of the chlorophylls of the antenna complex (DI_0_/RC), photosynthetic performance index (PI_ABS_), which incorporates the energy cascade processes from the first absorption events until the reduction of the plastoquinone (PQ), the maximum primary photochemical quantum yield (ϕ_P0_), the probability that a captured exciton will move an electron along the electron transporter chain (Ψ_E0_), and the quantum yield of electron transport (Φ_E0_).

The gas exchange was evaluated using an infrared gas analyzer (IRGA), model LI-6400XT (LI-COR Inc., Lincoln, USA). The IRGA was used to obtain the following parameters: net assimilation of CO_2_ (*A*), internal CO_2_ concentration (*Ci*), stomatal conductance of water vapor (*gsw*), and the transpiration rate (*E*). The data were always obtained from the youngest leaf that was fully expanded, and exposed to the sun. The measurements were obtained between 08:00 h and 11:00 h, using constant photosynthetically active radiation (PAR; 1000 μmol photons m^−2^ s^−1^), an atmospheric CO_2_ concentration of 400 µmol mol^−1^, a temperature of 25 °C, and relative humidity of 50% in the measurement chamber. The instantaneous efficiency of water use by the plants was calculated by the *A*/*E* ratio.

### 2.4. Growth

The percentage growth of the seedlings was calculated for the stem length (SL), stem diameter (SD), number of leaves (NL), maximum root length (RL), leaf area (LA), and total dry mass (TDM). The values were obtained by the equation:

$$x=\frac{100\left(b-a\right)}{a}$$

where:

*b* = the value recorded for the variable on the day of evaluation, and

*a* = the value recorded during the preceding evaluation.

### 2.5. Statistical Analyses

The data obtained for the different sampling intervals (30, 60, 90, 120, 150, and 180 DAT) were analyzed using a one-way ANOVA and linear regression. The regression models were selected based on the highest determination coefficients, and the significance of the regression coefficients was determined using the *t* test, with *p* < 0.05. The complete set of variables was evaluated together in a correlation matrix and combined in a principal components analysis (PCA). As the variables were measured in different units, a correlative PCA was applied, with the data being standardized to have a mean of 0 and standard deviation of 1. The number of components was selected according to the eigenvalues (>1.0) and the explained variance (>80%). The variables were also evaluated using Pearson’s correlation coefficient, with the strength of the relationship being evaluated by the *r* value, and the significance of the interaction being tested using a 5% probability threshold. All statistical analyses were run in the R environment, version 3.4.3. [41].

## 3. Results

### 3.1. Morpho-Anatomical Assessment

**Figure 1:**
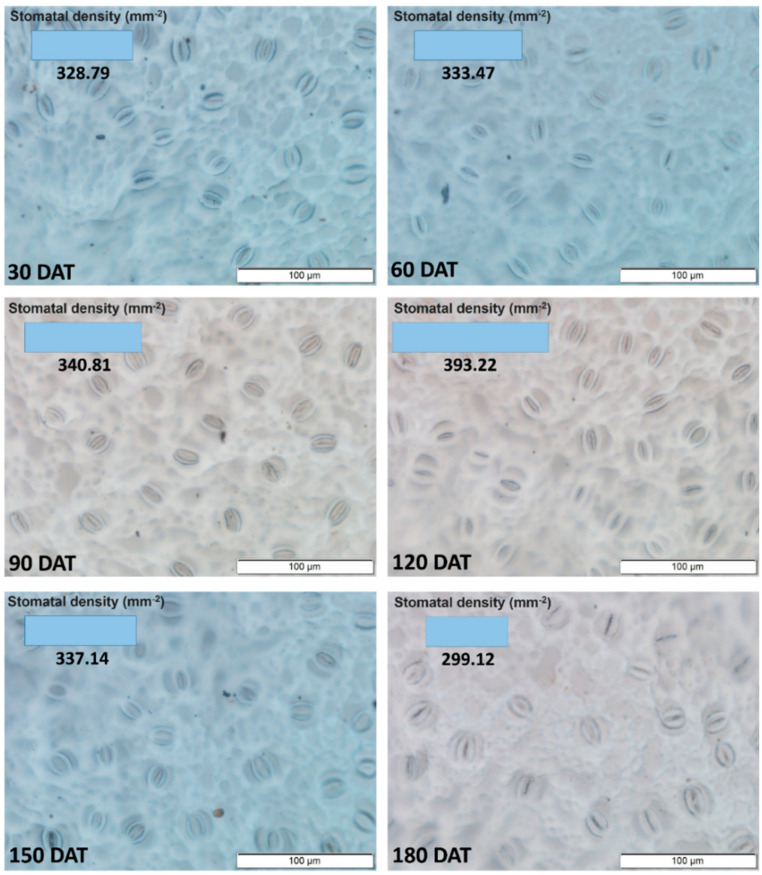
Density of stomata in the axial epidermis of the leaves of the seedlings of *Hymenaea courbaril* L., raised in a hydroponic system and sampled at different intervals of their initial development (30, 60, 90, 120, 150, and 180 days after transplantation).

The dispersal of the stomatal density data followed a quadratic function (Y = −0.0093 x^2^ + 1.8655x + 269.4; R^2^ = 60.00*), with the highest values being recorded for the leaves sampled at 120 DAT (Figure 1). The SD increased progressively during initial development up to 120 DAT, with the values decreasing progressively thereafter.

**Figure 2:**
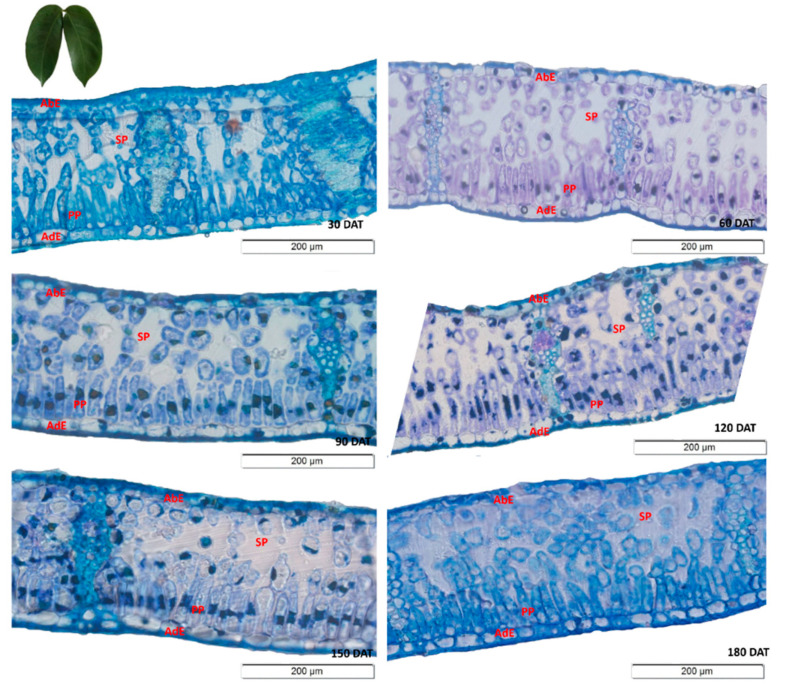
Transverse sections of the leaves of the seedlings of *Hymenaea courbaril* L. cultivated in a hydroponic system, showing the different anatomical regions (AbE = Abaxial Epidermis; AdE = Adaxial Epidermis; PP = Palisade Parenchyma; SP = Spongiform Parenchyma). Sections obtained at 30-day intervals across the initial development of the plants (30, 60, 90, 120, 150, and 180 days after transplantation).

As observed in the SD, the anatomical sections of the *H. courbaril* leaves revealed that the thickness of the palisade parenchyma increased over the first four sampling intervals (Figure 2). This parenchyma was thus thickest at 120 DAT, after which, the thickness decreased. Interestingly, as the spongiform parenchyma followed the exact opposite pattern, the data indicate that the *H. courbaril* plants compensated for the development of one of the parenchyma by reducing the other. This is supported by the fact that the thickness of the mesophyll did not vary systematically over the study period (Y = 0.0028x^2^ − 0.7752x + 223.88; R^2^ = 50.34*).

**Figure 3:**
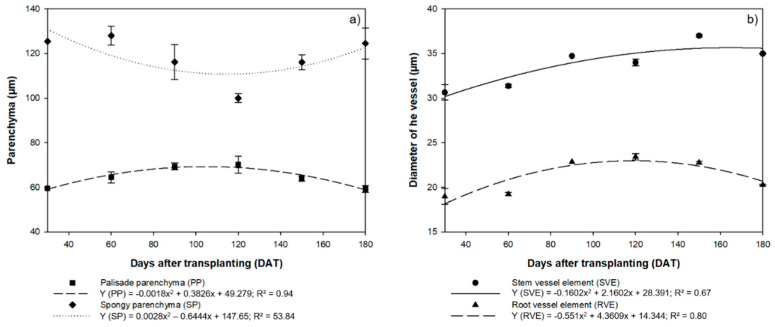
(**a**) Anatomical evaluation of the thickness of the Palisade Parenchyma (PP) and Spongiform Parenchyma (SP) of the seedlings of *Hymenaea courbaril* L. cultivated in the hydroponic system; (**b**) Diameter of the xylemic vessel elements in the stems (SVE) and roots (RVE) of the same plants. Sections obtained at 30-day intervals across the initial development of the plants (30, 60, 90, 120, 150, and 180 days after transplantation). Marker bars = SE (observed for eight repetitions).

The highest PP values (mean = 65.14 µm) were were thus recorded at 120 DAT, whereas the SP reached its lowest mean value (100.00 µm) at this stage. The contrasting investment by the *H. courbaril* plants in these two structures is clear from the inverse tendencies of the regression curves (Figure 3a).

The diameter of the stem xylemic vessels increased linearly in the initial phases, although there was a significant reduction at 180 DAT (34.97 µm), with the highest mean value (37.00 µm) being recorded at 150 DAT (Figure 3b). By contrast, the variation in the diameter of the root vessels (RVE) followed a pattern similar to that observed in the PP and SP, that is, the highest values (23.42 µm) were recorded at 120 DAT, with a reduction in this diameter in the subsequent intervals, that is, 22.76 µm at 150 DAT and 20.26 µm at 180 DAT (Figure 3b).

**Figure 4:**
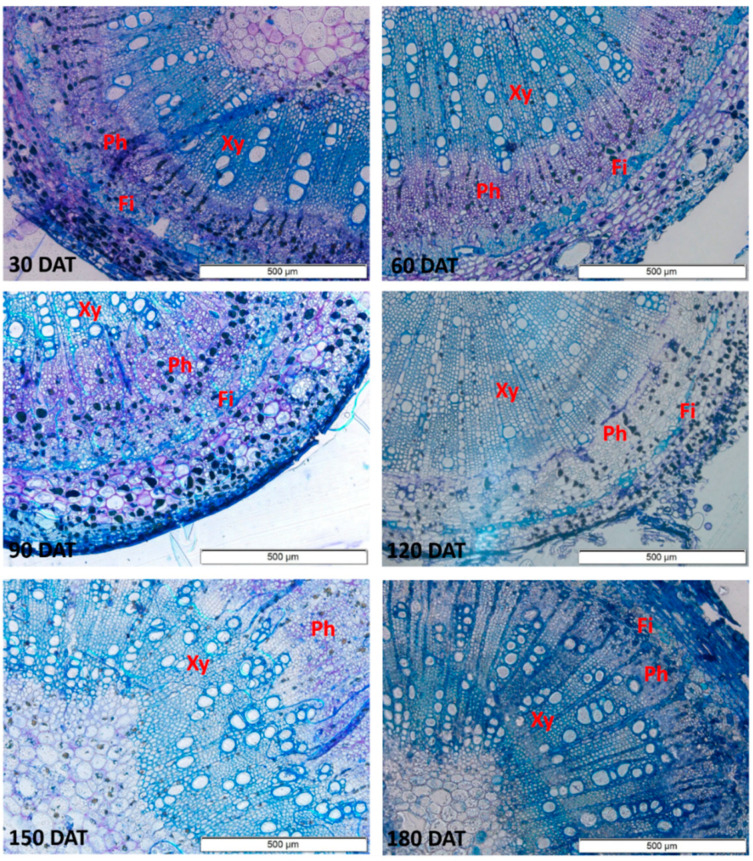
Sections of the stem of the seedlings of *Hymenaea courbaril* L. cultivated in a hydroponic system, showing the different anatomical features (Xy = Xylem; Ph = Phloem; Fi = Fibers). Sections obtained at 30-day intervals across the initial development of the plants (30, 60, 90, 120, 150, and 180 days after transplantation).

**Figure 5:**
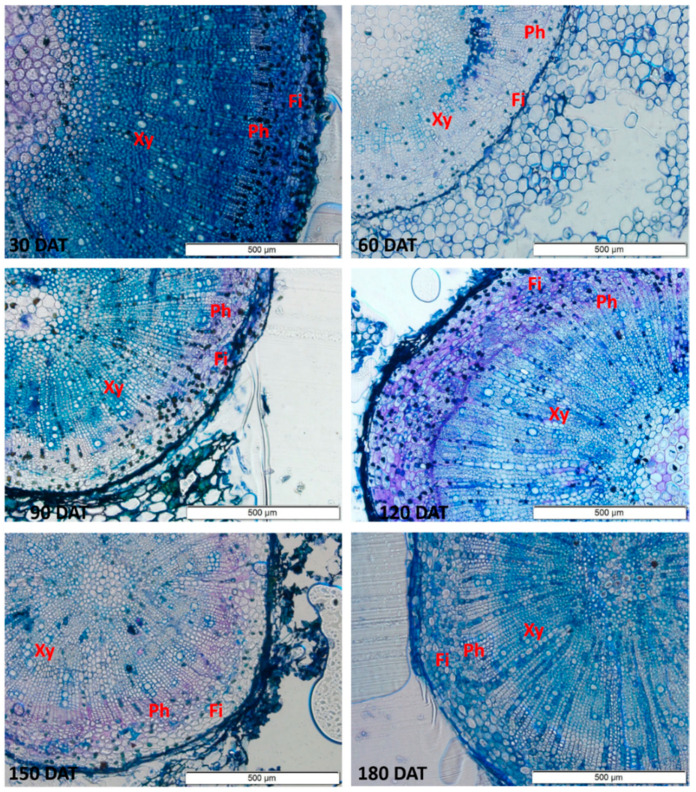
Sections of the root of the seedlings of *Hymenaea courbaril* L. cultivated in a hydroponic system, showing the different anatomical features (Xy = Xylem; Ph = Phloem; Fi = Fibers). Sections obtained at 30-day intervals across the initial development of the plants (30, 60, 90, 120, 150, and 180 days after transplantation).

The anatomical sections show vessels with larger diameters in the stem, which increased progressively up to 150 DAT (Figure 4). In the root (Figure 5), a progressive increase was observed up to 120 DAT, but the diameter subsequently declined significantly.

### 3.2. Physiological Assessment

**Figure 6:**
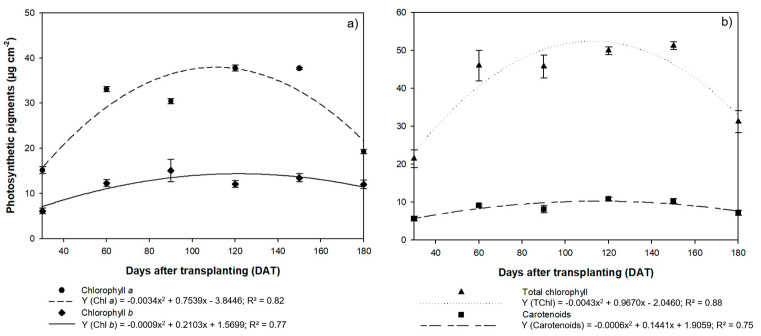
Mean concentrations of (**a**) chlorophyll *a* and *b* photosynthetic pigments and (**b**) total chlorophyll and carotenoids recorded in the leaves of the seedlings of *Hymenaea courbaril* L. cultivated in a hydroponic system and sampled at 30-day intervals across their initial development (30, 60, 90, 120, 150, and 180 days after transplantation). Marker bars = SE (Mean standard error - observed for eight repetitions).

The concentration of photosynthetic pigments in the *H. courbaril* plants also varied quadratically (Figure 6a,b). The highest mean Chla and carotenoid concentrations were recorded at 120 DAT and 150 DAT, with 37.76 µg cm^−2^ for Chla at 120 m DAT and 37.78 µg cm^−2^ at 150 DAT, and carotenoids at 10.83 µg cm^−2^ and 10.25 µg cm^−2^, respectively, with a drastic reduction at 180 DAT to 19.25 µg cm^−2^ for Chla and 7.12 µg cm^−2^ for carotenoids. The highest mean Chlb and TChl concentrations were also recorded at 150 DAT (13.48 µg cm^−2^ and 51.17 µg cm^−2^), followed, once again, by a reduction at 180 DAT, to 12.03 µg cm^−2^ for Chlb and 31.15 µg cm^−2^ for TChl.

**Figure 7:**
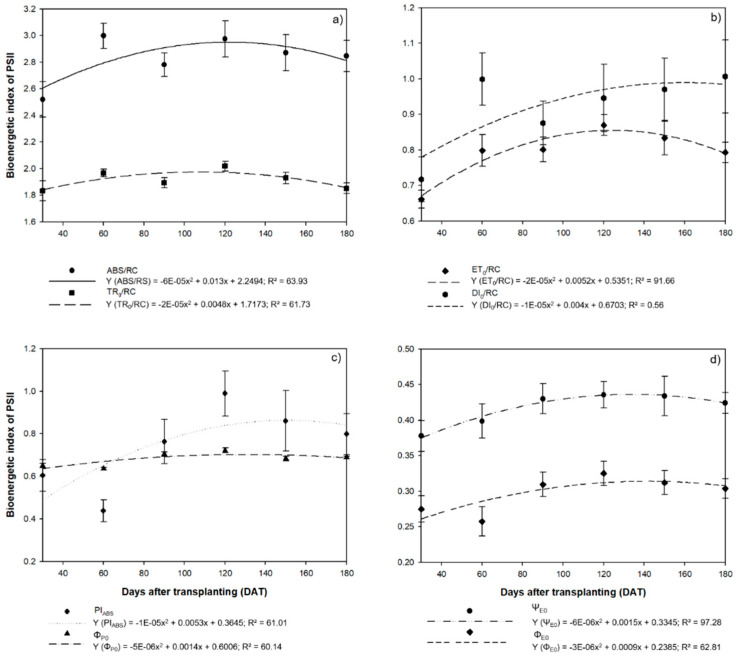
Parameters of chlorophyll *a* fluorescence observed in the leaves of the seedlings of *Hymenaea courbaril* L. cultivated in a hydroponic system and sampled at 30-day intervals across their initial development (30, 60, 90, 120, 150, and 180 days after transplantation): (**a**) the absorption flux energy per active reaction center (ABS/RC) and the energy flux captured by each reaction center (TR_0_/RC); (**b**) the electron transport flux per reaction center (ET_0_/RC) and the specific flux of the dissipation of energy at the level of the chlorophylls of the antenna complex (DI_0_/RC); (**c**) photosynthetic performance index (PI_ABS_) and the maximum primary photochemical quantum yield (ϕ_P0_); (**d**) the probability that a captured exciton will move an electron along the electron transporter chain (Ψ_E0_) and the quantum yield of electron transport (Φ_E0_). Marker bars = SE (observed for eight repetitions).

In the case of the parameters of chlorophyll *a* fluorescence, both the absorption flux energy (ABS/RC) and the energy flux captured by each reaction center (TR_0_/RC) tended to increase up to 120 DAT, when the ABS/RC was 2.97 and the TR_0_/RC was 2.02 (Figure 7a). These values subsequently decreased, with the ABS/RC reaching 2.87 at 150 DAT and 2.85 at 180 DAT, while the TR_0_/RC values reached 1.93 and 1.85 in the same two intervals. A similar trend was observed in the electron transport flux per reaction center (ET_0_/RC), with the highest mean value (0.87) being recorded at 120 DAT, subsequently decreasing to 0.83 at 150 DAT and 0.79 at 180 DAT (Figure 7b). However, the dissipation of energy (DI_0_/RC) tended to increase as the photosynthetic efficiency declined, reaching a value of 1.01 at 180 DAT.

Following a similar pattern to most of the other parameters described above, the best photosynthetic performance (PI_ABS_) was recorded at 120 DAT (0.99), and it declined subsequently to 0.86 at 150 DAT and 0.80 mat 189 DAT (Figure 7c). The maximum quantum yield (ϕ_P0_) also followed this general tendency, with the highest mean value (0.72) being recorded at 120 DAT. The probability that an exciton will move an electron along the transporter chain (Ψ_E0_, and the quantum yield of electron transport (Φ_E0_) both also reached a peak at 120 DAT (Ψ_E0_ = 0.44 and ϕ_E0_ = 0.33). Subsequently, both parameters declined, with ΨE_0_ reaching 0.68 at 150 DAT and 0.69 at 180 DAT, while the ϕ_E0_ values reached 0.43 at 150 DAT and 0.42 at 180 DAT (Figure 7d).

**Figure 8:**
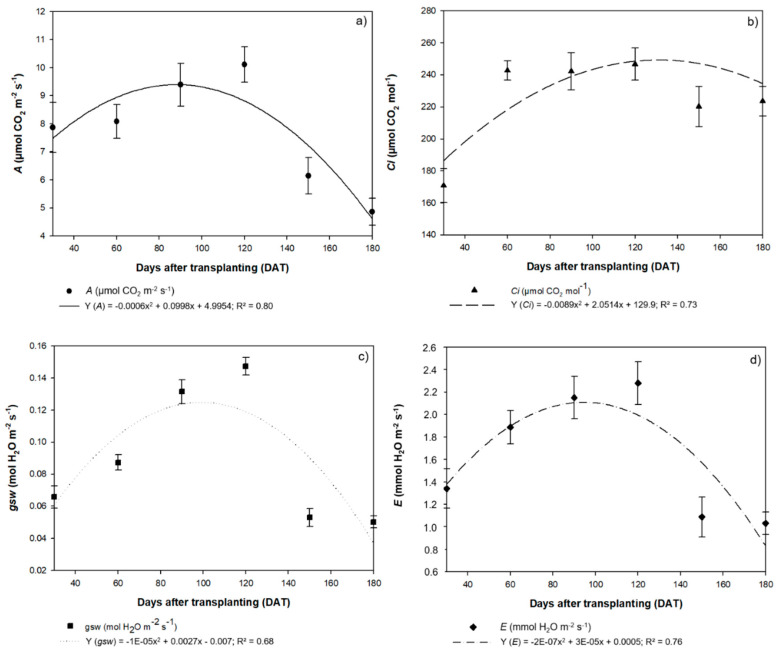
(**a**) Photosynthetic rate (*A*), (**b**) internal CO_2_ concentration (Ci), (**c**) stomatal conductance (*gsw*), and (**d**) transpiration rate (*E*) recorded in the seedlings of *Hymenaea courbaril* L. cultivated in a hydroponic system and sampled at 30-day intervals across their initial development (30, 60, 90, 120, 150, and 180 days after transplantation). Marker bars = SE (observed for eight repetitions).

The pattern of variation observed in the fluorescence data was also reflected in the photosynthetic rate (*A*), internal CO_2_ concentration (C*i*), stomatal conductance (*gsw*), and transpiration rate (*E*). These parameters also followed a quadratic model, and all peaked at 120 DAT, and subsequently decreased. The photosynthetic rate (*A*) peaked at 10.11 µmol of CO_2_ m^−2^ s^−1^, decreasing to 6.14 µmol of CO_2_ m^−2^ s^−1^ at 150 DAT and 4.87 µmol of CO_2_ m^−2^ s^−1^ at 180 DAT (Figure 8a). The C*i* concentrations reached a maximum of 246.7 µmol of CO_2_ m^−2^ s^−1^ at 120 DAT, declining to 220.2 µmol of CO_2_ m^−2^ s^−1^ at 150 DAT and 223.6 µmol of CO_2_ m^−2^ s^−1^ at 180 DAT (Figure 8b).

The highest stomatal conductance (*gsw*) was 0.15 mol of H_2_O m^−2^ s^−1^ at 120 DAT, with values declining to 0.05 mol of H_2_O m^−2^ s^−1^ at both 150 DAT and 180 DAT (Figure 8c). The highest transpiration rate (*E*) was 2.3 mmol de H_2_O m^−2^ s^−1^, recorded at 120 DAT (Figure 8d). At 150 DAT, *E* was 1.1 mmol de H_2_O m^−2^ s^−1^, while at 180 DAT, it was 1.0 mmol de H_2_O m^−2^ s^−1^. The high transpiration rate recorded at 120 DAT indicates that, despite the high photosynthetic rate, the plants were not using water efficiently during this period (*A*/*E* = 4.44 µmol de CO_2_ m^−2^ s^−1^/mmol de H_2_O m^−2^ s^−1^). This implies that the *H. courbaril* plants cultivated in a hydroponic system do not activate mechanisms to control transpiration, which would improve their photosynthetic efficiency. The *H. courbaril* plants used water more efficiently at 150 DAT (*A*/*E* = 5.65) and 180 DAT (*A*/*E* = 4.72), although, as they were in a hydroponic system, the reduction in the *E* values relative to *A* may have been related to anatomical alterations, such as a reduction in the SD and the diameter of the RVE.

### 3.3. Assessment of Growth and the Interactions among the Variables

**Figure 9:**
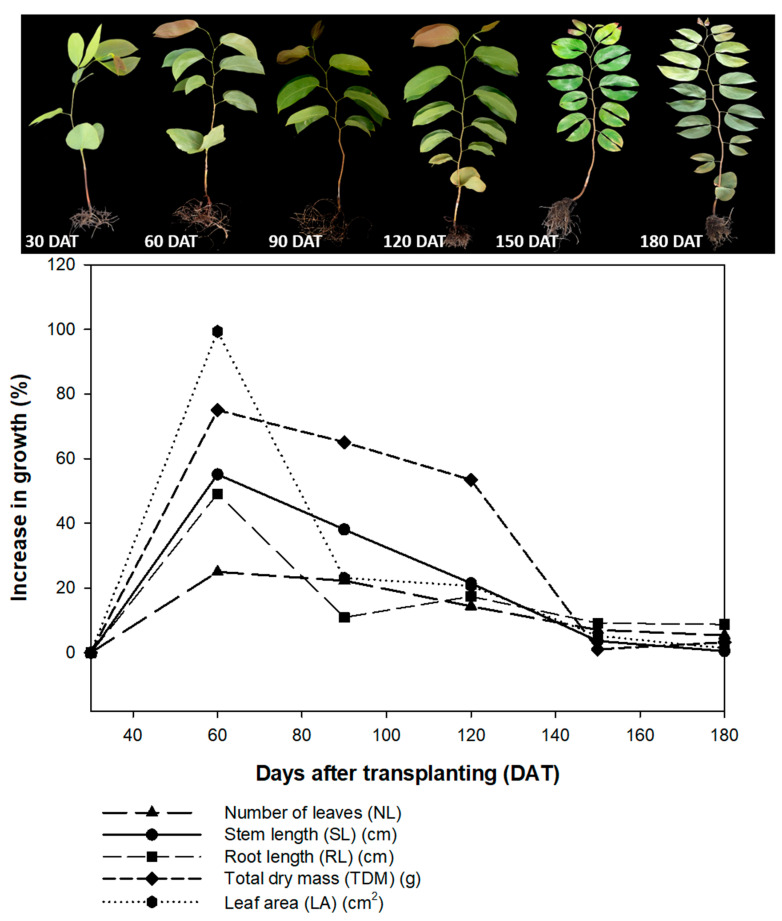
Percentage growth, number of leaves (NL); stem length (SL); root length (RL); total dry mass (TDM), and leaf area (LA) of the seedlings of *Hymenaea courbaril* L. cultivated in a hydroponic system and sampled at 30-day intervals across their initial development (30, 60, 90, 120, 150, and 180 days after transplantation).

All the parameters of plant growth reached their lowest values at 150 DAT and 180 DAT, indicating that, after 120 DAT, the hydroponic system may substantially alter the growth rates of the *H. courbaril* seedlings. The number de leaves for example, increased 3.63% between 120 and 150 DAT, and only 0.47% between 150 and 180 DAT, while stem length increased 7.00% between 120 and 150 DAT, and only 5.37% between 150 and 180 DAT. The length of the root increased 9.13% between 120 and 150 DAT, and only 8.71% between 150 and 180 DAT. The most significant reduction in growth rates were recorded in the leaf area and the total dry mass. Whereas leaf area increased 20.66% between 90 and 120 DAT, for example, it declined to 5.15% between 120 and 150 DAT, and to 1.39% between 150 and 180 DAT. Similarly, while dry mass increased 53.43% between 90 and 120 DAT, it subsequently increased only 1.01% by 150 DAT, and 3.21% by 180 DAT (Figure 9).

**Figure 10:**
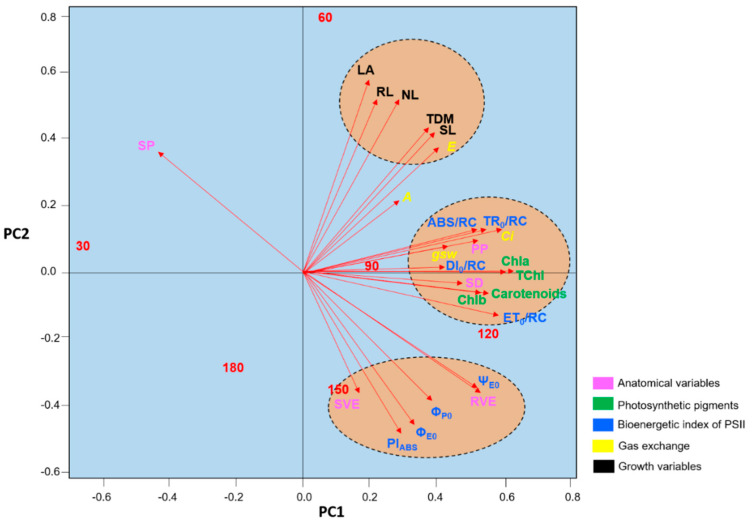
Plot of the results of the principal components analysis of the anatomical variables, photosynthetic pigments, chlorophyll *a* fluorescence parameters, gas exchange, and growth of the seedlings of *Hymenaea courbaril* L. cultivated in a hydroponic system and sampled at 30-day intervals across their initial development (30, 60, 90, 120, 150, and 180 days after transplantation).

The principal components analysis revealed three clusters of variables, with the growth parameters having similar weights in the determination of the differences observed among the evaluation periods (Figure 10). The concentrations of photosynthetic pigments, as well as the SD, ET_0_/RC, and DI_0_/RC, were important for the definition of the differences observed between 120 DAT and 90 DAT, while most of the parameters related to chlorophyll *a* fluorescence, together with the SVE and RVE, defined the differences between 150 DAT and 120 DAT. Clearly, 120 DAT was a period of transition between the lower values recorded at 90 DAT and 150 DAT.

The variation in the spongiform parenchyma (SP) followed a pattern opposite to that of the other variables, although this was expected, given its antagonistic relationship with the palisade parenchyma (PP), as observed in the regression model. The overall reduction in values recorded at 180 DAT, in particular those related to gas exchange (*A* and *E*), reduced the parameters to levels similar to those recorded at 30 DAT.

**Figure 11:**
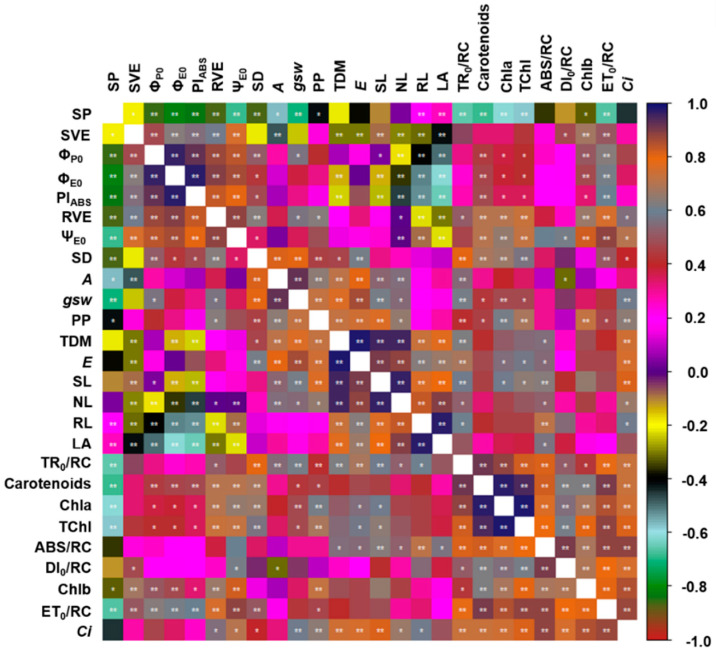
Correlation between the anatomical variables, photosynthetic pigments, chlorophyll *a* fluorescence parameters, gas exchange, and growth of the seedlings of *Hymenaea courbaril* L. cultivated in a hydroponic system and sampled at 30-day intervals across their initial development (30, 60, 90, 120, 150, and 180 days after transplantation). * significant at a probability of 5%; ** significant at a probability of 1%.

As expected, the correlation analysis showed that many of the chlorophyll *a* fluorescence parameters were correlated (Figure 11). Increases in ϕ_P0_, for example, were translated directly into an increase in the Ψ_E0_ (r = 0.96, *p* = 0.01) and the PI_ABS_ (r = 0.93, *p* = 0.01) values, while increasing PI_ABS_ values were reflected in an increase in the ϕ_E0_ (r = 0.80, *p* = 0.01). The parameters related to gas exchange were also correlated with the SD (SD vs. *A*: r = 0.82, *p* = 0.01; SD vs. *gsw*: r = 0.91, *p* = 0.01; SD vs. *E*: r = 0.61, *p* = 0.01). The *A* values were also correlated with the TDM (r = 0.72, *p* = 0.01), which confirms the importance of the plant’s photosynthetic activity for the total growth of the *H. courbaril* seedlings.

The growth parameters were also highly correlated with one another. The stem length (SL) was highly correlated with the NL (r = 0.97, *p* = 0.01), RL (r = 0.77, *p* = 0.01), and LA (r = 0.80, *p* = 0.01), indicating that the *H. courbaril* seedlings invested evenly in the growth of the different compartments of the plant. However, the concentrations of Chla were strongly correlated with the TChl (r = 0.98, *p* = 0.01), which indicates that the Chla concentration was the principal factor determining the levels of TChl found in the leaves of *H. courbaril*.

## 4. Discussion

### 4.1. Morpho-Anatomical Patterns

The SD, the thickness of the PP, and the diameter of the RVE all increased in the *H. courbaril* plants up to 120 DAT, although from this point onward, the plants began to reduce their investment in these structures. Plants cultivated hydroponically require large amounts of water each day; otherwise, if the input of fertilizers is not regulated adequately, the growing environment may become hypersalinated [42]. Given this, any reduction in the SD, which is reflected in a decrease in the transpirational flux (e.g., [43,44,45]), may help the plant to avoid the potentially damaging effects of high ion concentrations in hydroponic systems. A number of studies have shown that plants cultivated in these systems are more able to adapt to conditions of high salinity than plants cultivated using conventional methods [46], which may reflect a potential for the use of brackish water, or even seawater in hydroponic systems (e.g., [47,48,49,50]). This adaptation may be related directly to the anatomical and physiological adjustments observed in the *H. courbaril* seedlings in the present study.

We observed compensatory patterns in the two parenchyma evaluated in the *H. courbaril* seedlings. At 120 DAT, the plants had a thickened PP and a relatively thin SP. At 150 DAT and 180 DAT, however, as the volume of the PP in the leaf decreased, the volume of the SP increased. Growth also increased primarily up to 120 DAT, after which the size of the seedlings became incompatible with hydroponic cultivation, requiring large quantities of water, which meant that the pots in which they were cultivated became a limiting factor for their morpho-anatomical development. Pompelli et al. [51] demonstrated that the excessive availability of water reduces the spaces between the parenchymal cells, while a reduced availability of water reduces the thickness of the palisade layers. On the other hand, it seems clear that the roots of well-developed plants will compete for water, even in a hydroponic system. But as this demand is restricted by the size of the vessels, a reduction in the RVE diameter appears to be a good alternative for the reduction of the effects of this stress. Thangthong et al. [52] showed that plants under water stress have xylemic vessels of a smaller diameter and area. A reduction in the diameter of the xylemic vessels reduces the response to the high negative pressure generated by the transpiration of the leaves [53]. As we recorded the largest mean RVE diameter in the plants evaluated at 120 DAT, and thus the highest transpiration rate, the reduction in the RVE diameter observed at 150 DAT and 180 DAT, together with the reduction observed in the SD and PP, may be associated with the adjustments adopted by the plant to optimize the efficiency of its water use.

### 4.2. Physiological Patterns

The highest Chla concentrations in the leaves of the *H. courbaril* seedlings were recorded at 120 DAT, and are thus associated with the highest values recorded in the chlorophyll *a* fluorescence parameters in this period. The photosynthetic performance (PI_ABS_) peaked during this period, but declined during at 150 DAT and 180 DAT, which indicates that, in these final evaluations, some type of stress, such as competition for water, the salinization of the system, or limitations imposed by the size of the pots, was affecting the photosynthetic performance of the plants. Dai et al. [54] demonstrated that a reduction in the PI_ABS_ may reflect a decrease in the plant’s tolerance of the growing environment. Galić et al. [55] concluded that the PI_ABS_ can be used as a criterion for the selection of seeds with a high yield in heat-stressed environments, given that this index is more sensitive for the detection and quantification of damage, and that it relates the efficiency of the absorption, capture, and transfer of energy by photosystem II, which provides a better definition of the degree to which the stress of the environment affects the transportation of electrons [56].

Similarly, we observed a reduction in the flux of electrons per reaction center (ET_0_/RC) after 120 DAT, which also indicates a stress scenario, given that this flux is typically reduced in plants under stress from salinity or metals, for example (see [57,58,59]). By contrast, there was a progressive increase in the dissipation of energy in the form of heat (DI_0_/RC) at 150 DAT and 180 DAT, which is also consistent with the effects of stress on the plant. Under stress, a proportion of the reaction centers (DI_0_/RC) become dissipative centers to avoid photo-oxidative damage to the photosynthetic apparatus (e.g., [60,61]).

The *H. courbaril* plants also presented the highest *A*, *Ci*, and *gsw* values at 120 DAT, although this peak was associated with that in the SD. These values were also correlated, although an increase in the SD at 120 DAT was also associated with high transpiration rates, and thus a reduced efficiency in the use of water. The reduction of the SD is a long-term adaptation to a specific condition, and by altering the density and opening of its stomata, the plant is able to optimize its intake of CO_2_ for photosynthesis, while also minimizing water loss [62]. Dunn et al. [63] demonstrated that a moderate reduction in the SD of wheat plants (i.e., a reduction in density of less than 50%) did not alter yields in comparison with the control, but did result in an increase in the intrinsic efficiency of water use. Between 120 DAT and 150 DAT in the present study, the plants developed morpho-anatomical adaptations, such as a reduction in the SD and the diameter of the RVEs, that improve the efficiency of water use. This indicates that, despite being cultivated in a hydroponic system, the *H. courbaril* seedlings were not free of limitations, and were obliged to develop strategies to minimize water loss or to increase the availability of this resource in the growing pots, in order to reduce the possibility of saline damage due to the reduction of the amount of water in the vessels.

The linear increase observed in the *gsw* and *A* up to 120 DAT was associated with an increase in Chla and carotenoid concentrations. Carotenoids are pigments that are essential for photosynthesis, by absorbing the green-blue spectrum and transferring the absorbed energy to the chlorophylls, thus expanding the wavelengths available for photosynthesis [64]. The carotenoids also protect the plant from the potentially harmful effects of excessive exposure to sunlight [65]. The increase in *gsw* and *A* recorded at 120 DAT is also consistent with the growth of the plants up to this point. It seems reasonable to assume that an increase in the photosynthetic rate is translated directly into an increase in growth, although studies that have related photosynthesis and gas exchange systematically with primary growth are still scarce (e.g., [66]). Even so, the reduction observed in the *H. courbaril* growth rates in the intervals after 120 DAT supports the recommendation of not exceeding this period for the hydroponic cultivation of the seedlings of this plant species. Cruz et al. [45] concluded that cultivation systems which provoke a reduction in growth rates are inadequate, given that they tend to reduce survival rates.

The PCA also revealed that 120 DAT was the transition period between the lower mean values recorded at 90 DAT and 150 DAT, which supports our hypothesis that periods of cultivation of over 120 DAT may limit the growth of the *H. courbaril* seedlings raised in a hydroponic system. At 180 DAT, on the other hand, all the values recorded for the different study variables were more similar to those recorded at 30 DAT, which indicates that, after 120 DAT, the longer the seedlings are maintained in the hydroponic system, the greater the reductions in anatomical development, physiological activity, and growth rates. This allows us to also confirm the hypothesis that the observation of anatomical and physiological variables can provide reliable cues for the evaluation of the development of seedlings in hydroponic systems. This will guarantee that they are not maintained in the system after the peak in development, which will, in turn, ensure the most cost-effective application of the system to obtain seedlings.

## 5. Conclusions

We observed a progressive increase in the development of *H. courbaril* seedlings cultivated in a hydroponic system up to the 120th day after transplantation. After this period, however, the plants presented signs of stress, which indicated that 120 days would be the maximum period for the ideal maintenance of the seedlings of this species in the hydroponic system. We also confirmed the hypothesis that the morpho-anatomical and physiological responses of the hydroponic plants can predict the optimum period for transplantation, helping to minimize production time in this cultivation process.
